# Efficient parallel and out of core algorithms for constructing large bi-directed de Bruijn graphs

**DOI:** 10.1186/1471-2105-11-560

**Published:** 2010-11-15

**Authors:** Vamsi K Kundeti, Sanguthevar Rajasekaran, Hieu Dinh, Matthew Vaughn, Vishal Thapar

**Affiliations:** 1 Department of Computer Science and Engineering, University of Connecticut,371 Fairfield Way, U-2155, Storrs, CT, 06269, USA; 2 Texas Advanced Computing Center, University of Texas at Austin, TX, 78758, USA; 3 Cold Spring Harbor Laboratory, One Bungtown Road, Cold Spring Habor, NY, 11724, USA

## Abstract

**Background:**

Assembling genomic sequences from a set of overlapping reads is one of the most fundamental problems in computational biology. Algorithms addressing the assembly problem fall into two broad categories **- **based on the data structures which they employ. The first class uses an overlap/string graph and the second type uses a de Bruijn graph. However with the recent advances in short read sequencing technology, de Bruijn graph based algorithms seem to play a vital role in practice. Efficient algorithms for building these massive de Bruijn graphs are very essential in large sequencing projects based on short reads. In an earlier work, an *O*(*n/p*) time parallel algorithm has been given for this problem. Here *n *is the size of the input and *p *is the number of processors. This algorithm enumerates all possible bi-directed edges which can overlap with a node and ends up generating Θ(*n*Σ) messages (Σ being the size of the alphabet).

**Results:**

In this paper we present a Θ(*n/p*) time parallel algorithm with a communication complexity that is equal to that of parallel sorting and is not sensitive to Σ. The generality of our algorithm makes it very easy to extend it even to the out-of-core model and in this case it has an optimal I/O complexity of $\Theta (\frac{n\log(n/B)}{B\log(M/B)})$ (*M *being the main memory size and *B *being the size of the disk block). We demonstrate the scalability of our parallel algorithm on a SGI/Altix computer. A comparison of our algorithm with the previous approaches reveals that our algorithm is faster **- **both asymptotically and practically. We demonstrate the scalability of our sequential out-of-core algorithm by comparing it with the algorithm used by VELVET to build the bi-directed de Bruijn graph. Our experiments reveal that our algorithm can build the graph with a constant amount of memory, which clearly outperforms VELVET. We also provide efficient algorithms for the bi-directed chain compaction problem.

**Conclusions:**

The bi-directed de Bruijn graph is a fundamental data structure for any sequence assembly program based on Eulerian approach. Our algorithms for constructing Bi-directed de Bruijn graphs are efficient in parallel and out of core settings. These algorithms can be used in building large scale bi-directed de Bruijn graphs. Furthermore, our algorithms do not employ any all-to-all communications in a parallel setting and perform better than the prior algorithms. Finally our out-of-core algorithm is extremely memory efficient and can replace the existing graph construction algorithm in VELVET.

## Background

Sequencing genomes is one of the most fundamental problems in modern Biology and has immense impact on biomedical research. *De novo sequencing *is computationally more challenging when compared to sequencing with a reference genome. On the other hand the existing sequencing technology is not mature enough to identify/read the entire sequence of the genome **- **especially for complex organisms like the mammals. However small fragments of the genome can be read with acceptable accuracy. The *shotgun *sequencing employed in many sequencing projects breaks the genome randomly at many places and generates a large number of small fragments (*reads*) of the genome. The problem of reassembling all the fragmented reads into a small sequence close to the original sequence is known as the *Sequence Assembly *(SA) problem.

Although the SA problem seems similar to the *Shortest Common Super string *(SCS) problem, there are in fact some fundamental differences. Firstly, the genome sequence might contain several repeating regions. However, in any optimal solution to the SCS problem we will not be able to find repeating regions **- **because we want to minimize the length of the solution string. In addition to the repeats, there are other issues such as errors in reads and double strandedness of the reads which make the reduction to SCS problem very complex.

Existing algorithms to address the SA problem can be classified into two broad categories. The first class of algorithms model a read as a vertex in a directed graph **- **known as the *overlap graph *[1]. The second class of algorithms model every substring of length *k *(i.e., a *k*-mer) in a read as a vertex in a (subgraph of) a *de Bruijn *graph [2].

In an overlap graph, for every pair of overlapping reads, directed edges are introduced consistent with the orientation of the overlap. Since the transitive edges in the overlap graph are redundant for the assembly process they are removed and the resultant graph is called the *string graph *[1]. The edges of the string graph are classified into *optional*, *required *and *exact*. The SA problem is reduced to the identification of a shortest walk in the string graph which includes all the required and exact constraints on the edges. Identifying such a walk **- ***minimum S-walk*, on the string graph is known to be NP-hard [3].

In the de Bruijn graph model every substring of length *k *in a read acts as a vertex [2]. A directed edge is introduced between two *k*-mers if there exists some read in which these two *k*-mers overlap by exactly *k *- 1 symbols. Thus every read in the input is mapped to some path in the de Bruijn graph. The SA problem is reduced to a *Chinese Postman Problem *(CPP) on the de Bruijn graph, subject to the constraint that the resultant CPP tour include all the paths corresponding to the reads. This problem (*Shortest Super Walk*) is also known to be NP-hard. Thus solving the SA problem exactly on both of these graph models is intractable.

Overlap graph based algorithms were found to perform better (see [4-7]) with Sanger based read methods. Sanger methods produce reads typically around 1000 base pairs long and are very reliable. Unfortunately Sanger based methods are very expensive. To overcome the issues with Sanger reads, new read technologies such as sequencing by synthesis (Solexa) and pyrosequencing (454 sequencing) have emerged. These rapidly emerging read technologies are collectively termed as the *Next Generation Sequencing *(NGS) technologies. These NGS technologies can produce shorter genome fragments (anywhere from 25 to 500 base-pairs long). NGS technologies have drastically reduced the sequencing cost per base-pair when compared to Sanger technology. The reliability of NGS technology is acceptable, although it is relatively lower than the Sanger technology. In the recent past, the sequencing community has witnessed an exponential growth in the adoption of these NGS technologies.

On the other hand these NGS technologies can increase the number of reads in the SA problem by a large magnitude. The computational cost of building an overlap graph on these short reads is much higher than that of building a de Bruijn graph. De Bruijn graph based algorithms handle short reads very efficiently (see [8]) in practice compared to the overlap graph based algorithms. However the major bottleneck in using de Bruijn graph based assemblers is that they require a large amount of memory to build the de Bruijn graph.

This limits the applicability of these methods to large scale SA problems. In this paper we address this issue and present algorithms to construct large de Bruijn graphs very efficiently. Our algorithm is optimal in the sequential, parallel, and out-of-core models. A recent work by Jackson and Aluru [9] yielded parallel algorithms to build these de Bruijn graphs efficiently. They present a parallel algorithm that runs in *O*(*n/p*) time using *p *processors (assuming that *n *is a constant-degree polynomial in *p*). The *message complexity *of their algorithm is Θ(*n*Σ). By message complexity we mean the total number of messages (i.e., *k*-mers) communicated by all the processors in the entire algorithm. The distributed de Bruijn graph building algorithm in ABySS [10] is similar to the algorithm of [9].

One of the major contributions of our work is to show that we can build a bi-directed de Bruijn graph in Θ(*n/p*) time with a message complexity of Θ(*n*). An experimental comparison of these two algorithms on an SGI Altix machine shows that our algorithm is considerably faster. In addition, our algorithm works optimally in an out-of-core setting. In particular, our algorithm requires only $\Theta (\frac{n\log(n/B)}{B\log(M/B)})$ I/O operations.

## Methods

### Preliminaries

Let *s *∈ Σ*^n ^*be a string of length *n*. Any substring *s_j _*(i.e., *s*[*j*]*s*[*j *+ 1] . . . *s*[*j *+ *k *- 1], *n *- *k *+ 1 ≥ *j *≥ 1) of length *k *is called a *k-*mer of *s*. The set of all *k-*mers of a given string *s *is called the *k*-spectrum of *s *and is denoted by S (*s, k*). Given a *k*-mer *s_j _*, ${\bar{s}}_{j}$ denotes the *reverse complement *of *s_j _*(e.g., if *s_j _*= *AAGTA *then ${\bar{s}}_{j}=TACTT$). Let ≤ be the partial ordering among the strings of equal length such that *s_i _*≤ *s_j _*indicates that the string *s_i _*is lexicographically smaller than *s_j _*. Given any *k*-mer *s_i_*, let ${\hat{s}}_{i}$ be the lexicographically smaller string between *s_i _*and ${\bar{s}}_{j}$. We call ${\hat{s}}_{i}$ the *canonical k*-mer of *s_i_*. In other words, if $s_{i}\leq {\bar{s}}_{i}$ then ${\hat{s}}_{i}\;=s_{i}$ otherwise ${\hat{s}}_{i}\;={\bar{s}}_{i}$. A *k*-molecule of a given *k*-mer *s_i _*is a tuple $\left({\hat{s}}_{i},{\bar{\hat{s}}}_{i}\right)$ consisting of the canonical *k*-mer of *s_i _*and the reverse complement of the canonical *k*-mer. We refer to the reverse compliment of the canonical *k*-mer as *non-canonical k*-mer.

**Figure 1:**
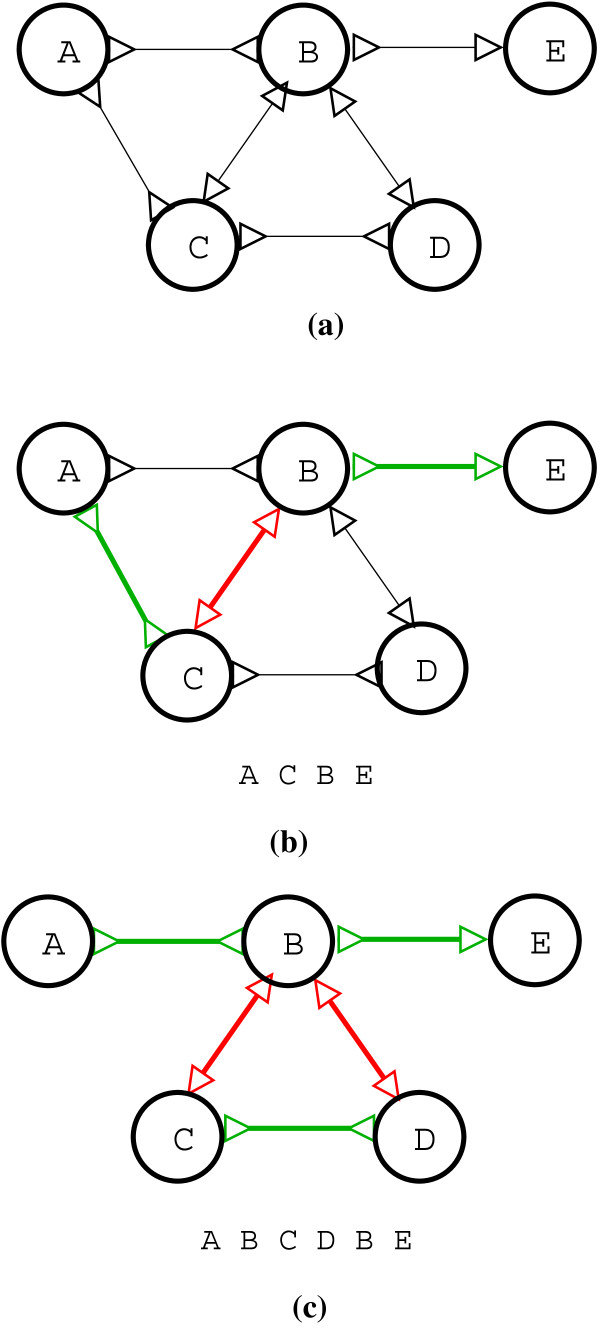
**This figure illustrates bi-directed between two nodes in a bi-directed graph**. (a) Shows a bi-directed graph with five nodes {*A, B, C, D, E*}. (b) The alternating green, red edges show a path between node *A *and *E*. (c) Shows two valid bi-directed walks from node *A *to *E*, the first path is {*A, B, E*} and the second path is {*A, B, C, D, B, E*}, this also shows that the bi-directed path may not be simple and can contain repeating nodes **- **in this case node *B*.

A *bi-directed *graph is a generalized version of a standard directed graph. In a directed graph every edge has only one arrow head (-▷ or ◁-). On the other hand in a bi-directed graph every edge has two arrow heads attached to it (◁-▷,◁-◁,▷-◁, or ▷-▷). Let *V *be the set of vertices and *E *= {(*v_i_, v_j _, o_1_, o*_2_)|*v_i_, v_j _*∈ *V *Λ *o_1_, o*_2 _∈ {◁, ▷}} be the set of bi-directed edges in a bi-directed graph *G*(*V, E*). For any edge *e *= (*v_i_, v_u_, o_1_, o*_2_) ∈ *E*, *o_1 _*= *e*[*o***^+^**] and *o*_2 _= *e*[*o^-^*] refer to the orientations of the arrow heads on the vertices *v_i _*and *v_j _*, respectively. A *walk W *(*v_i_, v_j _*) between two nodes *v_i_, v_j _*∈ *V *of a bi-directed graph *G*(*V, E*) is a sequence $v_{i},e_{i_{1}},v_{i_{1}},e_{i_{2}},v_{i_{2}},\ldots ,v_{i_{m}},e_{i_{m+1}},v_{j}$, such that for every intermediate vertex $v_{i}{}_{{}_{l}}$, 1 ≤ *l *≤ *m *the orientation of the arrow head on the incoming edge adjacent on $v_{i}{}_{{}_{l}}$ should match the orientation of the arrow head on the outgoing edge. To make this clearer, let $e_{i}{}_{{}_{l}},v_{i}{}_{{}_{l}},e_{i}{}_{{}_{l+1}}$ be a sub-sequence in the walk *W *(*v_i_, v_j _*). If $e_{i}{}_{{}_{l}}=(v_{i}{}_{{}_{l-1}},v_{i}{}_{{}_{l}},o_{1},\;o_{\text{2}})$ and $e_{i}{}_{{}_{l+1}}=(v_{i}{}_{{}_{l}},v_{i}{}_{{}_{l+1}},{o^{'}}_{1},{o^{'}}_{2})$ then for the walk to be valid it should be the case that $o_{\text{2}}={o^{'}}_{1}$. Figure 1(a) illustrates an example of a bi-directed graph. Figure 1(b) shows a simple bi-directed walk between the nodes *A *and *E*. A bi-directed walk between two nodes may not be simple. Figure 1(c) shows a bi-directed walk between *A *and *E *which is not simple **- **because *B *repeats twice.

A de Bruijn graph *D^k^*(*s*) of order *k *on a given string *s *is defined as follows. The vertex set *V *of *D^k^*(*s*) is defined as the *k*-spectrum of *s *(i.e., V=S(s, k)). We use the notation suf(*v_i_, l*) (*pre*(*v_i_, l*), respectively) to denote the suffix (prefix, respectively) of length *l *in the string *v_i_*. Let the symbol ◦ denote the concatenation operation between two strings. The set of directed edges *E *of *D^k^*(*s*) is defined as follows:

*E *= {(*v_i_, v_j _*)| suf(*v_i_, k *- 1) = *pre*(*v_j _, k *- 1) Λ *v_i_*[1] ◦ suf(*v_i_, k *- 1) ◦ *v_j _*[*k*] ∈ S(*s, k *+ 1)}. We can also define de Bruijn graphs for sets of strings as follows. If *S *= {*s_1_, s*_2 _. . . *s_n_*} is any set of strings, a de Bruijn graph *B^k^*(*S*) of order *k *on *S *has $V=\cup_{i=1}^{n}S(s_{i},k)$ and *E *= {(*v_i_, v_j _*)| suf(*v_i_, k *- 1) = *pre*(*v_j _, k *- 1) Λ ∃ *l *: *v_i_*[1] ◦ suf(*v_i_, k *- 1) ◦ *v_j _*[*k*] ∈ S(*s_l_, k *+ 1)}. To model the double strandedness of the DNA molecules we should also consider the reverse complements ($\bar{S}=\{{\bar{s}}_{1},{\bar{s}}_{2},\ldots ,{\bar{s}}_{n}\}$) while we build the de Bruijn graph.

To address this a bi-directed de Bruijn graph $BD^{k}(S\cup \bar{S})$ has been suggested in [3]. The set of vertices *V *of $BD^{k}(S\cup \bar{S})$ consists of all possible *k*-molecules from *S *∪ $\bar{S}$. The set of bi- directed edges for $BD^{k}(S\cup \bar{S})$ is defined as follows. Let *x, y *be two *k*-mers which are next to each other in some input string $z\in (S\cup \bar{S})$. Then an edge is introduced between the *k*- molecules *v_i _*and *v_j _*corresponding to *x *and *y*, respectively. Please note that two consecutive *k*-mers in some input string always overlap by *k *- 1 symbols. The converse need not be true. The orientations of the arrow heads on the edges are chosen as follows. If the canonical *k*-mers of nodes *v_i _*and *v_j _*overlap then an edge (*v_i_, v_j _*▷, ▷) is introduced. If the canonical *k*-mer of *v_i _*overlaps with the non-canonical *k*-mer of *v_j _*then an edge (*v*_*i*_, *v*_*j *_, ▷, ◁) is introduced. Finally if the non-canonical *k*-mer of *v_i _*overlaps with canonical *k*-mer of *v_j _*then an edge (*v*_*i*_, *v*_*j *_◁, ▷) is introduced.

**Figure 2:**
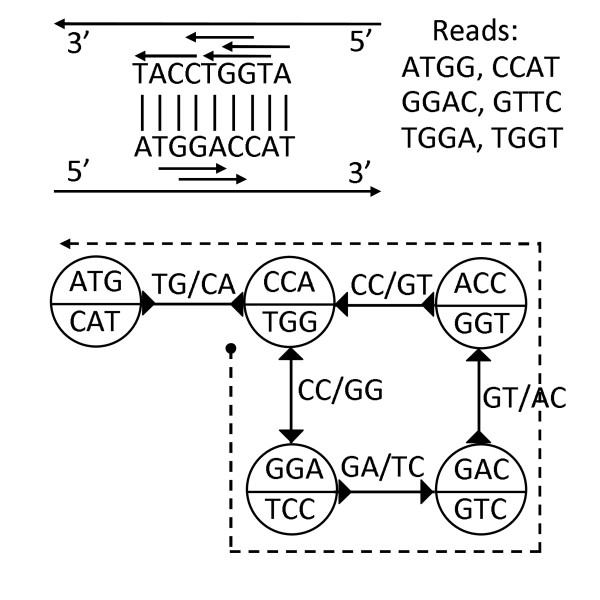
**A simple example of the bi-directed de Bruijn graph of order k = 3 from a set of reads ATGG,CCAT,GGAC,GTTC, TGGA and TGGT observed from a DNA sequence ATGGACCAT and its reverse complement ATGGTCCAT**. The dashed line shows one bi-directed walk which can reconstruct the original fragment.

Figure 2 illustrates a simple example of the bi-directed de Bruijn graph of order *k *= 3 from a set of reads *ATGG, CCAT, GGAC, GTTC, TGGA *and *TGGT *observed from a DNA sequence *ATGGACCAT *and its reverse complement *ATGGTCCAT*. Consider two 3-molecules *v_1 _*= (*GGA, TCC*) and *v_2 _*= (*GAC, GTC*). Because the canonical *k*-mer *x *= *GGA *in *v_1 _*overlaps with the canonical *k*-mer *y *= *GAC *in *v_2 _*by string *GA*, an edge (*v_1_, v_2_*, ▷, ▷) is introduced. Note that the non-canonical *k*-mer *GTC *in *v_2 _*also overlaps with the non-canonical *k*-mer *T CC *in *v_2 _*by string *T C*, so the two overlapping strings *GA *and *TC *are drawn above the edge (*v_1_, v_2_*, ▷, ▷) in Figure 2. A bi-directed walk on the example bi-directed de Bruijn graph as illustrated by the dashed line corresponds to the original DNA sequence with the first letter omitted *TGGACCAT*. We would like to remark that the parameter *k *is always chosen to be odd to ensure that the forward and reverse complements of a *k*-mer are not the same.

## Results

### Our algorithm to construct bi-directed de Bruijn graphs

In this section we describe our algorithm BiConstruct to construct a bi-directed de Bruijn graph on a given set of reads. The following are the main steps in our algorithm to build the bi-directed de Bruijn graph. Let *R_f _*= {*r*_1_, *r*_2 _. . . *r_n_*}, *r_i _*∈ Σ*^r ^*be the input set of reads. $\bar{R_{f}}=\{\bar{r_{1}},\bar{r_{2}}...\;\bar{r_{n}}\}$ is a set of reverse complements. Let $R*\;=R_{f}\cup {\bar{R}}_{f}$ and $R^{k}{}^{+\text{1}}=\cup_{r\in R^{*}}S\left(r,k+1\right)$. *R*^*k*+1 ^is the set of all (*k *+ 1)-mers from the input reads and their reverse complements.

• [STEP-1] **Generate canonical edges: **Let (*x, y*) = (*z*[1 . . . *k*], *z*[2 . . . *k *+ 1]) be the *k*-mers corresponding to a (*k *+ 1)-mer *z*[1 . . . *k *+ 1] ∈ *R*^*k***+1**^. Recall that $\hat{x}$ and *ŷ *are the canonical *k*- mers of *x *and *y*, respectively. Create a canonical bi-directed edge $\left({\hat{v}}_{i},{\hat{v}}_{j},o_{1},o_{2}\right)$ for each (*k *+ 1)-mer as follows.

$$(\hat{v_{i}},\hat{v_{j}},o_{1},o_{2})=\left\{ \begin{array}{l} \begin{array}{cc} \left(\hat{x},\hat{y},⊳,⊳\right) & \;\;\;\;x=\hat{x},y=\hat{y}\\ \left(\hat{y},\hat{x},⊲,⊲\right) & \begin{array}{l} \;\;\;\;\;\;\;\;\;\;\;\text{IF}\hat{x}\leq \hat{y},\\ \;\;\;\;\;\;\;\;\;\;\;\;\;\;\;\text{ELSE} \end{array} \end{array}\\ \begin{array}{cc} & x\neq \hat{x}\wedge y=\hat{y}\\ \left(\hat{x},\hat{y},⊲,⊳\right) & \;\;\;\;\;\;\;\;\;\;\text{IF}\;\hat{x}\leq \hat{y} \end{array}\\ \begin{array}{cc} \left(\hat{y},\hat{x},⊲,⊳\right) & \;\;\;\;\;\;\;\;\;\;\;\;\;\;\;\text{ELSE} \end{array}\;\\ \begin{array}{cc} & x=\hat{x}\wedge y\neq \hat{y}\\ \left(\hat{x},\hat{y},⊳,⊲\right) & \;\;\;\;\;\;\;\;\;\;\text{IF}\;\hat{x}\leq \hat{y} \end{array}\\ \begin{array}{cc} \left(\hat{y},\hat{x},⊳,⊲\right) & \;\;\;\;\;\;\;\;\;\;\;\;\;\;\;\text{ELSE} \end{array}\\ \begin{array}{cc} & x\neq \hat{x}\wedge y\neq \hat{y}\\ \left(\hat{x},\hat{y},⊲,⊲\right) & \;\;\;\;\;\;\;\;\;\text{IF}\;\hat{x}\leq \hat{y}, \end{array}\\ \begin{array}{cc} \left(\hat{y},\hat{x},⊳,⊳\right) & \;\;\;\;\;\;\;\;\;\;\;\;\;\;\text{ELSE} \end{array} \end{array}\right.$$

• [STEP-2] **Reduce multiplicity: **Sort all the bi-directed edges in [STEP-1], using radix sort. Since the parameter *k *is always odd this guarantees that a pair of canonical *k*-mers has exactly one orientation. Remove the duplicates and record the multiplicities of each canonical edge. Gather all the unique canonical edges into an edge list ℰ.

• [STEP-3] **Collect bi-directed vertices: **For each canonical bi-directed edge $({\hat{v}}_{i},{\hat{v}}_{j},o_{1},o_{2})\in ℰ$, collect the canonical *k*-mers ${\hat{v}}_{i}$, ${\hat{v}}_{j}$ into list V. Sort the list V and remove duplicates, such that V contains only the unique canonical *k*-mers.

• [STEP-4] **Adjacency list representation: **The list ℰ is the collection of all the edges in the bi-directed graph and the list V is the collection of all the nodes in the bi-directed graph. It is now easy to use ℰ and generate the adjacency lists representation for the bi-directed graph. This may require one extra radix sorting step.

### **Analysis of the algorithm **BiConstruct

**Theorem 1**. *Algorithm *BiConstruct *builds a bi-directed de Bruijn graph of order k in *Θ(*n*) *time. Here n is number of characters/symbols in the input*.

*Proof*. Without loss of generality assume that all the reads are of the same size *r*. Let *N *be the number of reads in the input. This generates a total of (*r *- *k*)*N *(*k *+ 1)-mers in [STEP-1]. The radix sort needs to be applied in at most 2*k *log(|Σ|) passes, resulting in 2*k *log(|Σ|)(*r *- *k*)*N *operations. Since *n *= *Nr *is the total number of characters/symbols in the input, the radix sort takes Θ(*kn *log(|Σ|)) operations assuming that in each pass of sorting only a constant number of symbols are used. If *k *log(|Σ|) = *O*(log *N *), the sorting takes only *O*(*n*) time. In practice since *N *is very large in relation to *k *and |Σ|, the above condition readily holds. Since the time for this step dominates that of all the other steps, the runtime of the algorithm BiConstruct is Θ(*n*).

### A parallel algorithm for building bi-directed de Bruijn graphs

In this section we illustrate a parallel implementation of our algorithm. Let *p *be the number of processors available. We first distribute *N/p *reads for each processor. All the processors can execute [STEP-1] in parallel. In [STEP-2] we need to perform parallel sorting on the list ℰ. Parallel radix/bucket sort **-**which does not use any all-to-all communications**- **can be employed to accomplish this. For example, the integer sorting algorithm of Kruskal, Rudolph and Snir takes $O\left(\frac{n}{p}\frac{\log n}{\log(n/p)}\right)$ time. This will be *O*(*n/p*) if *n *is a constant degree polynomial in *p*. In other words, for coarse-grain parallelism the run time is asymptotically optimal **- **which means optimality within a constant. In practice coarse-grain parallelism is what we have. Here *n *= *N *(*r *- *k *+ 1). We call this algorithm Par-BiConstruct.

**Theorem 2**. *Algorithm *Par-BiConstruct *builds a bi-directed de Bruijn graph in time O*(*n/p*). *The message complexity is O*(*n*).

The algorithm of Jackson and Aluru [9] first identifies the vertices of the bi-directed graph **- **which they call representative nodes. Then for every representative node |Σ| many-to-many messages are generated. These messages correspond to potential bi-directed edges which can be adjacent on that representative node. A bi-directed edge is successfully created if both the representatives of the generated message exist in some processor, otherwise the edge is dropped. This results in generating a total of Θ(*n*|Σ|) many-to-many messages. The authors in the same paper demonstrate that communicating many-to-many messages is a major bottleneck and does not scale well. We remark that the algorithm BiConstruct does not involve any many-to-many communications and does not have any scaling bottlenecks.

The algorithm presented in [9] can potentially generate spurious bi-directed edges. According to the definition [3] of the bi-directed de Bruijn graph in the context of SA problem, a bi-directed edge between two *k*-mers/vertices exists if and only if there exists some read in which these two *k*-mers are adjacent. We illustrate this by a simple example. Consider a read *r_i _*= *AATGCATC*. If we wish to build a bi-directed graph of order 3, then *AAT, ATG, TGC, GCA, CAT *, and *ATC *form a subset of the vertices of the bi-directed graph. In this example we see that the *k*-mers *AAT *and *ATC *overlap by exactly 2 symbols. However there cannot be any bi-directed edge between them according to the definition **- **because they are not adjacent. On the other hand the algorithm presented in [9] generates the following edges with respect to the *k*-mer *AAT *: {(*AAT, ATA*), (*AAT, ATG*), (*AAT, ATT *), (*AAT, ATC*)}. The edges (*AAT, ATA*) and (*AAT, ATC*) are purged since the *k*-mers *ATA *and *ATC *are missing. However bi-directed edges with corresponding orientations are established between *ATG *and *ATC*. Unfortunately (*AAT, ATC*) is a spurious edge and can potentially generate wrong assembly solutions. In contrast to their algorithm [9] our algorithm does not use all-to-all communications **- **although we use point-to-point communications.

### Out of core algorithms for building bi-directed de Bruijn graphs

**Theorem 3**. *There exists an out-of-core algorithm to construct a bi-directed de Bruijn graph using an optimal number of I/O***'***s*.

*Proof*. Replace the radix sorting with an external *R*-way merge sort which takes only $\Theta (\frac{n\log(n/B)}{B\log(M/B)})$ I/O**'**s. Here *M *is the main memory size, *n *is the sum of the lengths of all reads, and *B *is the block size of the disk.

### Simplified bi-directed de Bruijn graphs

**Figure 3:**
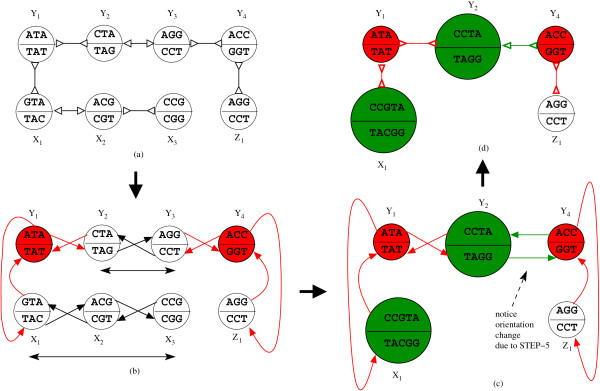
**The red nodes in Figure 3(b) indicate the nodes in the set *V*' (STEP-1), similar red colored edges indicate the edges in *E*'**. After list ranking (STEP-3) we will have four maximal chains as follows, $\left(Y_{2}^{-},Y_{3}^{+}),\;(Y_{3}^{-},Y_{2}^{+}),\;(X_{1}^{-},X_{2}^{+},X_{3}^{-})(X_{3}^{+},X_{2}^{-},X_{1}^{+}\right)$. Now if we stick to the convention described in STEP-5 we renumber the new node corresponding to the chains $\left(Y_{2}^{-},Y_{3}^{+}),\;(Y_{3}^{-},Y_{2}^{+}\right)$ as *Y_2_*. As a result the edges $\left(Y_{3}^{+},Y_{4}^{-}\right)$ and $\left(Y_{4}^{+},Y_{3}^{-}\right)$ are updated (shown in green in Figure 3(c)) as $\left(Y_{2}^{-},Y_{4}^{-}\right)$ and $\left(Y_{4}^{+},Y_{2}^{+}\right)$ Finally the directed edges are replaced with bi-directed edges in Figure 3(d).

The bi-directed de Bruijn graph constructed in the previous section may contain several linear chains. These chains have to be compacted to save space as well as time. The graph that results after this compaction step is referred to as the *simplified bi-directed graph*. A linear chain of bi-directed edges between nodes *u *and *v *can be compacted only if we can find a valid bi-directed walk connecting *u *and *v*. All the *k*-mers/vertices in a compactable chain can be merged into a single node, and relabelled with the corresponding forward and reverse complementary strings. In Figure 3 we can see that the nodes *X*_1 _and *X*_3 _can be connected with a valid bi-directed walk and hence these nodes are merged into a single node. In practice the compaction of chains plays a very crucial role. It has been reported that merging the linear chains can reduce the number of nodes in the graph by up to 30% [8].

**Figure 4:**
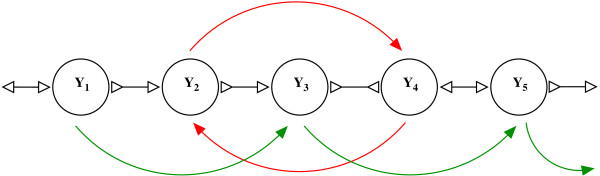
**An example illustrating problems with pointer jumping on bi-directed chains**. The green colored arcs indicate valid pointer jumps, however the red colored arcs create cyclic loops and will create problems in the next pointer jumping operation.

Although the bi-directed chain compaction problem seems like a *list ranking *problem there are some fundamental differences. Firstly, a bi-directed edge can be traversed in both the directions. As a result, applying *pointer jumping *directly on a bi-directed graph can lead to cycles and cannot compact the bi-directed chains correctly. Figure 4 illustrates the first phase of pointer jumping. Pointer jumping is an operation on a directed chain/linked list which changes the neighbour of a list node to its neighbour's neighbour. As we can see, the *green *arcs indicate valid pointer jumps from the starting nodes. However since the orientation of the node *Y*_4 _is reverse relative to the direction of pointer jumping a cycle results. In contrast, a valid bi-directed chain compaction would merge all the nodes between *Y*_1 _and *Y*_5 _since there is a valid bi-directed walk between *Y*_1 _and *Y*_5_. On the other hand, bi-directed chain compaction may result in changing the orientation of some bi-directed edges and these edges should be recognised and updated accordingly. Consider a bi-directed chain in Figure 3(a). This chain contains two possible bi-directed walks **- ***Y*_2 _to *Y*_3 _and *X*_1 _to *X*_3_, see Figure 3(b). The walk from *Y*_3 _to *Y*_2 _(*Y*_2 _to *Y*_3_) spells out a label *TAGG*(*CCTA*) after compaction. Once we perform this compaction (see Figure 3(c)) the orientation of the edge between *Y*_3 _and *Y*_4 _in the original graph is no longer valid, because the label *CCTA *on the newly compacted node cannot overlap with the label *GGT *on the node *Y*_4_. However the label *TAGG *on the newly compacted node overlaps with label *GGT *on the *Y*_4 _and hence its orientation should be updated. On the other hand after the edge between *X*_1 _and *Y*_1 _does not need any update after compacting the nodes *X*_1_, *X*_2 _and *X*_3_, see Figure 3(b) and Figure 3(c).

**Figure 5:**
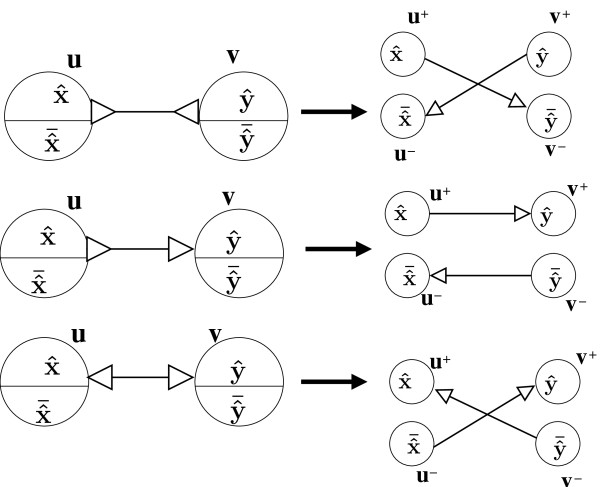
**An illustration of the ListRankingTransform which replaces every bi-directed edge with a pair of directed edges as shown here**.

Since bi-directed chain compaction has a lot of practical importance, efficient and correct algorithms are essential. We now provide algorithms for the bi-directed chain compaction problem. Our key idea here is to transform a bi-directed graph into a directed graph and then apply *list ranking*. We define the ListRankingTransform as an operation which replaces every bi-directed edge with a pair of directed edges with some orientation **- **see Figure 5 for these orientations.

Given a list of candidate canonical bi-directed edges, we apply a ListRankingTransform (see Figure 5) which introduces two new nodes *v***^+^**, *v***^- ^**for every node *v *in the original graph. Directed edges corresponding to the orientation are introduced. See Figure 5.

**Lemma 1**. *Let BG*(*V, E*) *be a bi-directed graph; let BG^t^*(*V^t^, E^t^*) *be the directed graph after applying *ListRankingTransform. *Two nodes u, v *∈ *V are connected by a bi-directed path iff u***^+ ^**∈ *V^t ^(u***^- ^**∈ *V^t^) is connected to one of v***^+^***(v***^-^***) or v***^-^***(v***^+^***) by a directed path*.

**Figure 6:**
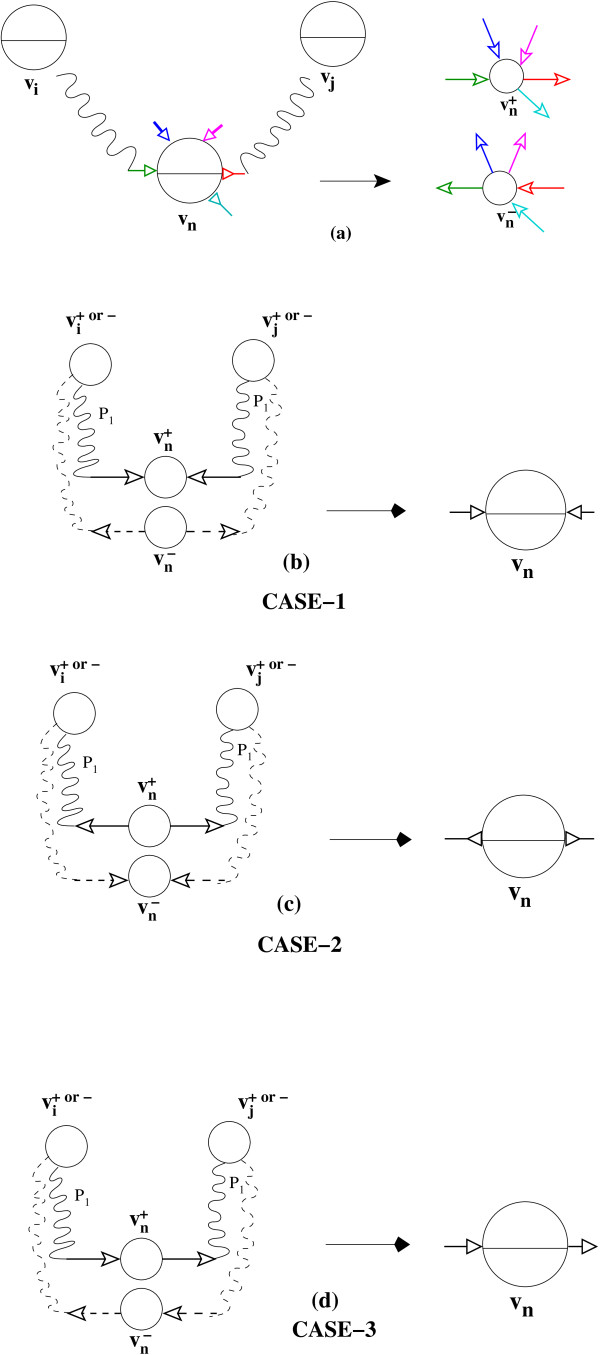
**Proof that ListRankingTransform preserves bi-directed walk in the original graph**.

*Proof*. We first prove the forward direction by induction on the number of nodes in the bi- directed graph. Consider the *basis *of induction when |*V *| = 2, let *v*_0_, *v*_1 _∈ *V*. Clearly we are only interested when *v*_0 _and *v*_1 _are connected by a bi-directed edge. By the definition of ListRankingTransform the Lemma in this case is trivially true. Now consider a bi-directed graph with |*V *| = *n *+ 1 nodes, if the path between *v_i_, i < n *and *v_j _, j < n *does not involve node *v_n _*the lemma still holds by induction on the sub bi-directed graph *BG*(*V *- {*v*_n_}, *E*). Now assume that *v_i _. . . v_p_, v_n_, v_q _. . . v_j _*is the bi-directed path between *v_i _*and *v_j _*involving the node *v_n_*. (See Figure 6(a)). Figure 6(a) shows what the transformed directed graph looks like. Observe the colors of bi-directed edges and the corresponding directed edges. By the induction hypothesis on the sub bi-directed paths *v_i _. . . v_p_, v_n _*and *v_n_, v_q _. . . v_j _*we have the following. $v_{i}^{+}$ is connected to $v_{n}^{+}$ or $v_{n}^{-}$ by some directed path *P*_1 _(See Figure 6(b)); $v_{n}^{+}$ is connected to $v_{j}^{+}$ or $v_{j}^{-}$ by some directed path *P*_2_. We examine three possible cases depending on how the directed edge from *P*_1 _and *P*_2 _is incident on $v_{n}^{+}$ In CASE-1 we have both *P*_1 _and *P*_2 _pointing into node $v_{n}^{+}$. This implies that the orientation of the bi-directed edges in the original graph is according to Figure 6(b). In this case we cannot have a bi-directed walk involving the node *v_n_*, which contradicts our original assumption. Similarly CASE-2 (Figure 6(c)) would also lead to a similar contradiction. Only CASE-3 would let node *v_n _*be involved in a bi-directed walk. In this case $v_{i}^{+}$ will be connected to either $v_{j}^{+}$ or $v_{j}^{-}$ by concatenation of the paths *P*_1 _and *P*_2_. We can make a similar argument to prove the reverse direction.

#### *Algorithm for bi-directed chain compaction*

Given a bi-directed graph we now give an outline of the algorithm which compacts all the valid bi-directed chains.

• STEP-1: Apply the ListRankingTransform for each bi-directed edge. Let the resultant directed graph be *G*(*V, E*).

• STEP-2: Identify a subset of nodes *V*' = {*v *: *v *∈ *V *, (*d*_*i*n_(*v*) *>*1 or *d*_o*u*t_(*v*) *>*1)} and a subset of edges *E*' = {(*u, v*) : (*u, v*) ∈ *E*, (*u *∈ *V*' or *v *∈ *V*')}.

• STEP-3: Apply pointer jumping on the directed graph *G*(*V *- *V*', *E *- *E*').

• STEP-4: Now let $\left(v_{i}^{+},\ldots ,v_{j}^{-}\right)$ be a maximal chain obtained after pointer jumping. Due to the symmetry in the graph there exists a corresponding complementary chain $\left(v_{j}^{+},\ldots ,v_{i}^{-}\right)$. Each chain is replaced with a single node and its label is the concatenation of all the labels in the chain. We should stick to some convention while we give a new number to the newly created node. For example, we can choose min{*v_i_, v_j _*} as the new number for the newly created node. In our example if *v_i _*= min{*v_i_, v_j _*} then we replace the chain $\left(v_{i}^{+},\ldots ,v_{j}^{-}\right)$ with node $v_{i}^{+}$ and relabel it with the concatenated label. Similarly, the chain $\left(v_{j}^{+},\ldots ,v_{i}^{-}\right)$ will be replaced with the node $v_{i}^{-}$ and relabeled accordingly.

• STEP-5: Finally, to maintain the connectivity we need to update the edges in *E*' to reflect the changes during compaction. Coming back to our example, we have replaced the chain $\left(v_{i}^{+},\ldots ,v_{j}^{-}\right)$ with the node $v_{i}^{+}$. As a result we need to replace any edge $(x,v_{j}^{-})\in E'$ with the edge $\left(x,v_{i}^{+}\right)$. Similarly we also need to update any edges adjacent on $v_{j}^{+}$.

Note that all of the above steps can be accomplished with some constant number of radix sorting operations. Figure 3 illustrates the compaction algorithm on a bi-directed graph. The red nodes in Figure 3 indicate the nodes in the set *V*'. Red colored edges indicate the edges in *E*'. After list ranking we will have four maximal chains as follows: $\left(Y_{2}^{-},Y_{3}^{+}),\;(Y_{3}^{-},Y_{2}^{+}),\;(X_{1}^{-},X_{2}^{+},X_{3}^{-})(X_{3}^{+},X_{2}^{-},X_{1}^{+}\right)$. Now if we stick to the convention described in STEP-5 we renumber the new node corresponding to the chains $\left(Y_{2}^{-},Y_{3}^{+}),\;(Y_{3}^{-},Y_{2}^{+}\right)$ as *Y*_2_. As a result the edges $\left(Y_{3}^{+},Y_{4}^{-}\right)$ and $\left(Y_{4}^{+},Y_{3}^{-}\right)$ are updated (shown in green in Figure 3(c)) as $\left(Y_{2}^{-},Y_{4}^{-}\right)$ and $\left(Y_{4}^{+},Y_{2}^{+}\right)$. Finally the directed edges are replaced with bi-directed edges in Figure 3(d).

#### Analysis of bi-directed compaction on parallel and out-of-core models

Let ℰ*_l _*be the list of candidate edges for compaction. To do compaction in parallel, we can use a *Segmented Parallel Prefix *[11] on *p *processors to accomplish this in time $O(|2{ℰ}_{l}|/p+\log(p))$. List ranking can also be done out-of-core as follows. Without loss of generality we can treat the input for the list ranking problem as a set *S *of ordered tuples of the form (*x, y*). Given *S *we create a copy and call it *S'*. We now perform an external sort of *S *and *S' *with respect to *y *(i.e., using the *y *value of tuple (*x, y*) as the key) and *x*, respectively. The two sorted lists are scanned linearly to identify tuples (*x, y*) ∈ *S*, (*x*',*y*') ∈ *S' *such that *y *=*x*'. These two tuples are merged into a single tuple (*x, y*') and is added to a list ${ℰ}_{l}{}^{'}$. This process is now repeated on ${ℰ}_{l}{}^{'}$. Note that if the underlying graph induced by ℰ*_l _*does not have any cycles then $|{ℰ}_{l}^{'}|\leq |{ℰ}_{l}|/2$; which means that the size of ${ℰ}_{l}{}^{'}$ geometrically decreases after every iteration. The I/O complexity of each iteration is dominated by the external sorting and hence bi-directed compaction can be accomplished out-of-core with $\Theta (\log_{2}(C)\frac{\left|{ℰ}_{l}\right|}{B}\log_{\frac{M}{B}}(\frac{\left|{ℰ}_{l}\right|}{B}))$ I/O operations, where *C *is the length of the longest chain. Care should be taken to deal with cycles. The sort-merge algorithm mentioned above will run forever in the presence of cycles. To address this we can maintain what is known as *join count *in every sort-merge phase. Given any *S *the *join count *of a tuple (*x, y*) ∈ *S *is an indicator variable *J*(*x, y*) = 1, if ∃(*y, z*) ∈ *S*; else 0. Finally *J*(*S*) = Σ_(*x*, *y***)**∈**S **_*J*(*x, y*). Notice that the function *J*(*S*) strictly decreases in every sort and merge phase and finally becomes zero when there are no cycles. However in the presence of cycles the function *J*(*S*) decreases and then remains constant. Thus if the function *J*(*S*) remains constant in any two consecutive sort-merge phases we can stop iterating and report that there are some cycles. Once we stop the list ranking we can easily detect the edges in the cycles. Our implementation of this out-of-core list ranking based on this idea is available at http://trinity.engr.uconn.edu/~vamsik/ex-list-rank.

### Improving the construction of the bi-directed de Bruijn graph in some practical assemblers

**Figure 7:**
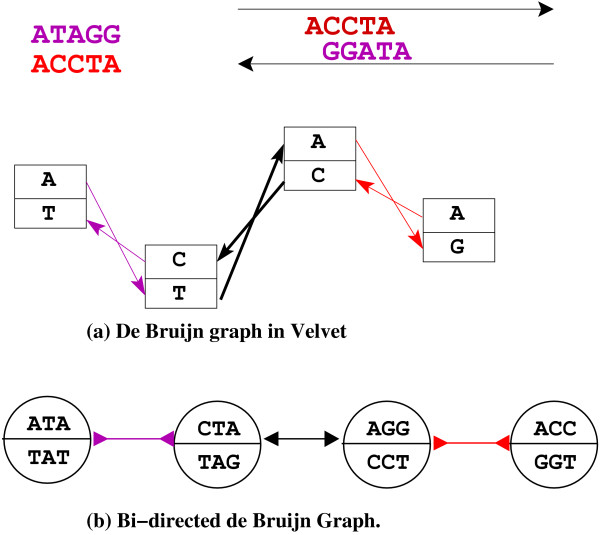
**This figure illustrates the subtle differences between internal graph representation of velvet and the bi-directed graph**. (a) de Bruijn graph representation in velvet for the read fragment above, note velvet keeps track of only the first amino-acid symbol from the forward and reverse compliments. (b) bi-directed graph representation for the same read fragment.

In this section we briefly describe how our algorithms can be used to speedup some of the existing SA programs. As an example, we consider VELVET [8]. VELVET is a suite of programs **- **velveth and velvetg, which has recently gained acclamation in assembling short reads. The VELVET program builds a simplified bi-directed graph from a set of reads. We now briefly describe the algorithm used in VELVET to build this graph. The VELVET program puts all the *k*-mers from the input into a hash table and then identifies the *k*-mers which are present in at least 2 reads **- **this information is called the *roadmap *in VELVET**'**s terminology. The program then builds a de Bruijn graph using these *k*-mers. Finally it takes every read and threads it on these *k*-mers. The worst case time complexity is *O*(*n *log(*n*)) **- **assuming that the implementation of hash table is based on a balanced search tree. However VELVET uses a Splay tree so this would be the amortized runtime rather than the worst case. Since VELVET builds this graph entirely in-memory, this has some serious scalability problems especially on large scale assembly projects. However VELVET has some very good assembly heuristics to remove errors and identify redundant assembly paths, etc. Our out-of-core algorithm can act as a replacement to the code in VELVET that performs in-memory graph construction. The internal de Bruijn graph of VELVET is slightly different from the bi-directed graph our algorithm builds. It is easy to see the equivalence between these two representations. (See Figure 7). We have implemented an out-of-core algorithm that takes in a file with reads and the value of *k *and generates the graph for VELVET program. To be more precise, VELVET program creates a file with the name Graph in the directory when we run the velvetg program. We have modified the code in the VELVET program by adding an option which quits after it builds the Graph file without any simplification. This gives us a chance to compare the VELVET**'**s algorithm which can build the Graph file with our algorithm. The results and more details about the program are in the results section.

## Discussion

Before we go into the discussion we briefly describe our experimental setting. We used a SGI-Altix 64-bit, 64 node supercomputer with a RAM of 2 GB per node for our parallel algorithm experiments. For our sequential out-of-core experiments we used a 32-bit x86 machine with 1 GB of RAM. All our algorithms are implemented in C under Linux environment.

We have compared the performance of our algorithm and that of Jackson and Aluru [9]. We refer to the later algorithm as JA. To make this comparison fair, we have implemented the JA algorithm (because their implementation is unavailable) also on the same platform that our algorithm runs on. We have used the SGI/Altix IA-64 machine for all of our experiments. Our implementation uses MPI for communication between the processors. We used a test set of 8 million un-paired reads obtained from sequencing a plant genome at CSHL. The performance of both the algorithms is measured with various values of *k *(the de Bruijn graph parameter) on multiple processors. Both the algorithms (JA and ParBiConstruct) use the same underlying parallel sorting routines.

Table 1 shows the user and system times for both our algorithm and the JA algorithm. We can clearly see that the system time (communication time) is consistently higher for the JA algorithm. Also notice that as we increase the value of *k *keeping the number of processors fixed both the algorithms become faster. On the other hand ParBiConstruct is consistently superior than the JA algorithm for all the parameters. Also notice the speedup (time taken by the JA algorithm divided by the time taken by ParBiConstruct) of ParBiConstruct in Table 1. We obtain a maximum speedup of 24.9*X *on the real time when *k *= 21 and *p *= 64. This is clearly because the communication cost is very high and the JA algorithm enumerates all the possible overlaps. Finally, over all the settings for *k *and *p *ParBiConstruct is around 8*X *faster than the JA algorithm with a maximum speedup of 25*X *when *k *= 21, *p *= 64.

**Table 1:** Comparison of runtime between the JA algorithm and our algorithm on an input with 8 million reads from a plant genome

		JA ALGO			ParBiConstruct					
**P**	**k**	**U(sec)**	**S(sec)**	**R(mm:ss)**	**U(sec)**	**speedup**	**S(sec)**	**speedup**	**R(mm:ss)**	**Speedup**

8	21	6388.08	21.54	13:26.42	1028.61	6.21	8.08	2.67	2:10.77	6.17

8	27	2220.41	14.85	5:47.71	500.22	4.44	3.72	3.99	1:04.12	5.42

8	33	1662.14	6.63	3:29.89	288.14	5.77	2.47	2.68	0:37.46	5.60

8	35	496.89	3.81	1:03.77	159.59	3.11	1.03	3.70	0:21.19	3.01

12	21	9116.72	20.31	12:43.14	985.97	9.25	6.91	2.94	1:23.95	9.09

12	27	2626.30	13.94	3:59.85	479.10	5.48	3.20	4.36	0:41.32	5.80

12	33	2320.07	6.14	3:15.15	317.08	7.32	2.35	2.61	0:27.76	7.03

12	35	561.02	3.47	0:48.19	166.06	3.38	1.13	3.07	0:15.04	3.20

16	21	11943.18	19.91	12:29.49	1044.31	11.44	7.42	2.68	1:06.91	11.20

16	27	2889.32	12.95	3:50.81	498.36	5.80	3.42	3.79	0:32.48	7.11

16	33	2971.37	6.49	3:07.41	357.63	8.31	2.53	2.57	0:23.64	7.93

16	35	580.08	3.67	0:37.63	170.56	3.40	1.33	2.76	0:11.85	3.18

24	21	17744.55	20.26	12:21.94	1205.51	14.72	8.00	2.53	0:51.74	14.34

24	27	3399.06	15.24	2:39.02	658.02	5.17	4.00	3.81	0:28.72	5.54

24	33	4981.96	7.89	6:25.41	652.80	7.63	3.69	2.14	0:51.65	7.46

24	35	750.26	5.57	0:37.30	295.58	2.54	2.23	2.50	0:14.19	2.63

32	21	23119.80	20.95	12:04.89	1070.31	21.60	8.40	2.49	0:34.90	20.77

32	27	3897.63	15.20	2:12.96	464.24	8.40	5.21	2.92	0:15.81	8.41

32	33	5132.44	9.33	3:11.64	534.38	9.60	4.84	1.93	0:21.01	9.12

32	35	973.78	5.35	0:37.30	324.23	3.00	3.35	1.60	0:13.16	2.83

48	21	37116.65	26.37	13:03.67	2422.83	15.32	13.46	1.96	0:55.33	14.16

48	27	4932.47	21.70	1:48.76	1112.72	4.43	10.51	2.06	0:26.11	4.17

48	33	6658.10	13.72	3:11.84	1157.33	5.75	9.41	1.46	0:34.33	5.59

48	35	1020.88	10.95	0:30.47	447.84	2.28	8.18	1.34	0:14.08	2.16

64	21	51443.09	35.46	16:25.33	1938.25	26.54	25.05	1.42	0:39.57	24.90

64	27	6304.66	33.49	2:05.96	1029.89	6.12	21.80	1.54	0:21.64	5.82

64	33	15314.25	23.95	5:57.82	673.55	22.74	21.31	1.12	0:17.08	20.95

64	35	1048.56	20.71	0:25.93	358.62	2.92	18.86	1.10	0:09.87	2.63

Input Reads: 8388608 short reads, each of size 36 base pairs

Average User time speed up = 8.310X

Average System time speed up = 2.490X

Average Real time speed up = 8.079X

The user time of our algorithm is also consistently superior compared to the user time of JA. This clearly is because we do much less local computations. In contrast, JA needs to do a lot of local processing, which arises from processing all the received edges, removing redundant ones, and collecting the necessary edges to perform many-to-many communications.

We also compare the memory used by both the algorithms. We briefly describe how we obtained the memory reports in our experiments. Since the memory used by each processor is different during execution at any given instance we add-up the memory used by each processor and divide by the number of processors and we report this number in our experiments. We obtained the resident memory and shared memory from the top command and averaged it over the number of memory probe samples obtained by top. Table 2 gives details of the memory usage of both the algorithms. From these results it is clear that our approach is also efficient compared to the JA algorithm. ParBiConstruct uses upto 4*X *less resident memory compared to the JA algorithm.

**Table 2:** Comparison of memory between the JA algorithm and our algorithm on an input with 8 million reads from a plant genome.

		JA ALGO		ParBiConstruct			
**P**	**k**	**RES.MEM(Mb)**	**SHR.MEM(Mb)**	**RES.MEM(Mb)**	**efficiency**	**SHR.MEM(Mb)**	**efficiency**

8	21	903.714	3.690	244.603	3.695	3.328	1.109

8	27	675.516	3.638	146.750	4.603	3.232	1.126

8	33	289.787	3.588	62.580	4.631	3.007	1.193

8	35	116.938	7.527	14.625	7.996	2.397	3.140

12	21	681.664	4.782	156.875	4.345	4.256	1.124

12	27	265.304	6.882	91.219	2.908	3.889	1.769

12	33	241.630	4.721	46.805	5.163	3.675	1.285

12	35	94.548	7.518	12.276	7.702	2.771	2.713

16	21	501.175	5.726	152.995	3.276	5.175	1.107

16	27	333.487	11.730	77.523	4.302	4.823	2.432

16	33	166.395	5.714	42.746	3.893	4.628	1.235

16	35	68.851	8.172	13.578	5.071	3.443	2.374

24	21	344.888	8.081	105.723	3.262	7.133	1.133

24	27	214.681	16.271	61.674	3.481	6.690	2.432

24	33	115.630	7.830	34.583	3.344	5.959	1.314

24	35	52.110	10.082	15.990	3.259	4.472	2.254

32	21	260.063	10.413	86.157	3.018	9.070	1.148

32	27	179.657	20.550	49.737	3.612	8.096	2.538

32	33	95.790	9.968	34.180	2.803	7.495	1.330

32	35	47.602	11.290	15.289	3.113	4.905	2.302

48	21	186.225	14.109	71.073	2.620	12.405	1.137

48	27	112.237	19.623	47.596	2.358	11.255	1.744

48	33	75.059	11.996	30.929	2.427	9.070	1.323

48	35	37.500	10.643	19.576	1.916	6.764	1.573

64	21	150.324	17.311	63.449	2.369	14.640	1.182

64	27	105.692	23.570	43.590	2.425	12.975	1.817

64	33	66.095	14.168	31.853	2.075	10.653	1.330

64	35	38.823	12.159	22.275	1.743	8.096	1.502

Input Reads: 8388608 short reads, each of size 36 base pairs

Average Efficiency of RESident memory across all processors = 3.622

Average Efficiency of SHaRed memory across all processors 1.667

Since JA takes a significant amount of time for inputs larger than 8 million, we have compared these algorithms only for input sizes up to 8 million. The experimental results reported in [9] start with at least 64 processors. We however show the scalablity of our algorithm for up to 128 million (randomly generated) reads in Table 3. Table 3 clearly demonstrates the scalability of our algorithm. We make our implementations and all the details of test cases used available at http://trinity.engr.uconn.edu/~vamsik/ParBiDirected.

**Table 3:** Scalability of our algorithm for up to 128 million reads. Since our test dataset contained only 8 million reads, we generated these reads randomly, each read was of size 35 and *k *= 33 was used.

reads	p	user time (ticks)	sys time (ticks)	wall time (min:sec)
16777216	2	37147	259	1:14.02

33554432	2	148070	1219	2:42.66

67108864	2	340653	2348	6:18.77

134217728	2	770922	5560	15:00.42

16777216	4	37254	85	0:38.95

33554432	4	99067	677	1:48.60

67108864	4	240861	1931	4:14.57

134217728	4	471196	4272	8:29.62

16777216	8	20217	57	0:21.90

33554432	8	47319	322	0:55.41

67108864	8	153782	1781	2:39.18

134217728	8	314281	3456	5:17.65

16777216	16	16951	55	0:19.73

33554432	16	17936	135	0:25.64

67108864	16	64408	804	1:10.91

134217728	16	135562	2148	2:21.83

16777216	32	12901	40	0:16.38

33554432	32	9973	191	0:17.55

67108864	32	46659	486	0:53.32

134217728	32	82414	950	1:28.87

### Out-of-core algorithm versus VELVET graph building algorithm

Our aim is to study the computational efficiency of the current VELVET**'**s algorithm to build the de Bruijn graph and our algorithm. To accomplish this we have modified the code of VELVET to stop once it completes building the graph from the reads. This is done as follows. Firstly we run the velveth program to complete the building of RoadMaps. The code of velvetg is modified such that the program dumps out the Graph file after threading of the reads. Our out-of-core algorithm generates Graph file directly by taking the reads file and the value of *k*. We have used a low end desktop 32-bit machine with 1 GB RAM to demonstrate the scalability of our out-of-core algorithm. Our results indicate that the VELVET algorithm starts *virtual memory trashing *[12] for around 4 million reads with *k *= 21. This trashing leads to massive increase in the page-faults and stalls the program from progressing further. Thus the VELVET algorithm cannot build large bi-directed graphs. In contrast to VELVET our algorithm works with a constant (user specified) amount of memory and scales well for building large amounts of reads **- **which we demonstrate in Table 4.

**Table 4:** Comparison of our algorithm with VELVET on a 32-bit machine with 1 GB of RAM

	OUT OF CORE				VELVET	
**reads**	**initial** **edges**	**edges after** **multiplicity reduction and canonicalization**	**page faults**	**time** **hrs:min:sec**	**page faults**	**Time** **hrs:min:sec**

2097152	31457280	21387750	5593	00:10:31	82	00:4:31

4194304	62914560	32443128	23084	00:22:40	2419455	07:50:02*

6291456	94371840	39460652	40920	00:34:09	1255816	04:22:28*

8388608	125829120	44840055	45716	00:38:13	1064952	03:50:02*

* Indicates that VELVET program did not complete and the program was stopped after this time.

The program is available at http://trinity.engr.uconn.edu/~vamsik/ex-build-vgraph/.

## Conclusions

In this paper we have presented an efficient algorithm to build a bi-directed de Bruijn graph, which is a fundamental data structure for any sequence assembly program **- **based on an Eulerian approach. Our algorithm is also efficient in parallel and out of core settings. These algorithms can be used in building large scale bi-directed de Bruijn graphs. Also, our algorithm does not employ any all-to-all communications in a parallel setting and performs better than that of [9]. Finally, our out-of-core algorithm can build these graphs with a constant amount of RAM, and hence can act as a replacement for the graph construction algorithm employed by VELVET [8].

## Authors' contributions

VK designed and implemented the algorithms, and drafted the manuscript. SR participated in designing the algorithms, revised the manuscript, and coordinated all phases of the project. HD also participated in the design of algorithms and contributed towards the figures in the manuscript. MV and VT introduced the sequence assembly problem to the team and provided short read data to validate our method. All the authors read and approved the final manuscript.

## References

[B1] Kececioglu JD Myers EW Combinatorial algorithms for DNA sequence assembly Algorithmica 1995 13 1-2 7 51 10.1007/BF01188580

[B2] Pevzner PA Tang H Waterman MS An Eulerian path approach to DNA fragment assembly Proceedings of the National Academy of Sciences of the United States of America 2001 98 17 9748 9753 10.1073/pnas.171285098 11504945 PMC55524

[B3] Medvedev P Georgiou K Myers G Brudno M Computability of models for sequence assembly Workshop on Algorithms for Bioinformatics (WABI), LNBI-4645 2007 289 301 full_text

[B4] Huang X Wang J Aluru S Yang S Hillier L PCAP: A whole-genome assembly program Genome research 2003 13 9 2164 2170 http://www.scopus.com [Cited By (since 1996): 61] 10.1101/gr.1390403 12952883 PMC403719

[B5] Myers EW Sutton GG Delcher AL Dew IM Fasulo DP Flanigan MJ Kravitz SA Mobarry CM Reinert KHJ Remington KA Anson EL Bolanos RA Chou H Jordan CM Halpern AL Lonardi S Beasley EM Brandon RC Chen L Dunn PJ Lai Z Liang Y Nusskern DR Zhan M Zhang Q Zheng X Rubin GM Adams MD Venter JC A whole-genome assembly of Drosophila Science 2000 287 5461 2196 2204 10.1126/science.287.5461.2196 10731133

[B6] Batzoglou S Jaffe DB Stanley K Butler J Gnerre S Mauceli E Berger B Mesirov JP Lander ES ARACHNE: A whole-genome shotgun assembler Genome research 2002 12 177 189 10.1101/gr.208902 11779843 PMC155255

[B7] PHRAP ASSEMBLER http://www.phrap.org/

[B8] Zerbino DR Birney E Velvet: Algorithms for de novo short read assembly using de Bruijn graphs Genome research 2008 18 5 821 829 10.1101/gr.074492.107 18349386 PMC2336801

[B9] Jackson BG Aluru S Parallel construction of bidirected string graphs for genome assembly International Conference on Parallel Processing 2008 346 353 full_text

[B10] Simpson JT Wong K Jackman SD Schein JE Jones SJM Birol I ABySS: A parallel assembler for short read sequence data Genome research 2009 19 6 1117 1123 10.1101/gr.089532.108 19251739 PMC2694472

[B11] Jaja J Introduction to Parallel Algorithms Addison Wesley

[B12] Silberschatz A Baer P Gagne G Operating System Princples Wiley

